# 360-degree virtual reality video to teach neonatal resuscitation: an exploratory development study

**DOI:** 10.1038/s41598-024-65299-4

**Published:** 2024-06-22

**Authors:** Sevag Tachejian, Ahmed Moussa

**Affiliations:** 1 Division of Pediatric Emergency Medicine, Department of Pediatrics, The Hospital for Sick Children, Toronto, ON Canada; 2 Division of General Pediatrics, Department of Pediatrics, CHU Sainte-Justine, Montreal, QC Canada; 3 Division of Neonatology, Department of Pediatrics, CHU Sainte-Justine, Montreal, QC Canada; 4 CHU Sainte-Justine Research Center, Montreal, QC Canada; 5 Centre for Applied Health Sciences Education (CPASS), Faculty of Medicine, Université de Montréal, Montreal, QC Canada

**Keywords:** Neonatology, Software

## Abstract

Simulation is an effective training method for neonatal resuscitation (NR). However, the limitations brought about by the COVID-19 pandemic, and other resource constraints, have necessitated exploring alternatives. Virtual reality (VR), particularly 360-degree VR videos, have gained attraction in medical training due to their immersive qualities. The primary objectives of the study were to produce a high quality 360-degree virtual reality (VR) video capturing NR simulation and to determine if it could be an acceptable adjunct to teach NR. The secondary objective was to determine which aspects of NR could benefit from the incorporation of such a video in training. This was an exploratory development study. The first part consisted of producing the video using a GoPro action camera, Adobe Premiere Pro, and Unity Editor. In the second part participants were recruited, based on level of experience, to watch the video and answer questionnaires to determine acceptability (user experience and cognitive load) and aspects of NR which could benefit from the video. The video was successfully developed. Forty-six participants showed a strong general appreciation. User experience revealed high means (> 6) in the positive subscales and low means (< 4) for immersion side effect, with no difference between groups. Cognitive load was higher than anticipated. Participants indicated that this video could be effective for teaching crisis resource management principles, human and environment interactions, and procedural skills. The 360-degree VR video could be a potential new simulation adjunct for NR. Future studies are needed to evaluate learning outcomes of such videos.

## Introduction

Every year, thousands of neonates born in North America need resuscitation, from airway management with positive pressure ventilation to endotracheal intubation and cardiac massage^1^. These skills are most often acquired through the Neonatal Resuscitation Program (NRP) training in North America. In Canada, NRP training begins with a self-directed learning period (textbook and completion of an online exam), followed by an in-person session consisting of practical skills stations, simulation training, and an evaluation that leads to certification^2^.

Simulation has been demonstrated as an effective method to teach neonatal resuscitation (NR), acute crisis resource management, teamwork, and procedural skills^3–8^. Deliberate practice of procedural skills, without involving patients, coupled with feedback and debriefing sessions allow for better assessment of the students’ skills and better performance^3–8^. However, in person simulation has numerous limitations resource-wise: time, cost of material and of support personnel, and duty-hour regulations^4,9,10^, and it can also lead to wasteful use of medical supplies^8^. Furthermore, the COVID-19 pandemic with its sanitary measures concerning in-person teaching sessions has forced restructuration of simulations^11–14^.

As accessibility increased, a keen interest has been given to immersive technologies overseen by virtual reality (VR), especially in the procedural medical fields^15–17^. VR is defined as a computer-generated simulation of a real-life situation^18^. Augmented reality (AR) encompasses layers of computer-generated enhancements to an existing reality to make the experience more interactive^18^. The development of camera technologies has given lieu to the production of 360-degree videos, which offer a unique immersive experience, when projected using head mounted devices (HMDs) (referred from here on as 360-degree VR videos). 360-degree VR videos allow for independent learning, and unlimited number of opportunities to practice teamwork, communication, and patient management skills. The learner can gain familiarity with the environment at his own pace, before real life exposure^10,12^.

Snelson and Hsu, in a systematic review, concluded that 360-degree VR videos might be more appropriate for promoting empathy, reflection, or skill-based knowledge as opposed to factual or conceptual knowledge^19^. Pirker and Dengel, in a multidisciplinary literature review, showed that 360-degree VR videos can benefit learning processes in terms of performance, motivation, and knowledge retention. Moreover, the impacts of 360-degree VR videos in medicine and healthcare are more appreciable because of their high standard of realism and the fact that shocking experiences can be cushioned^11^. These videos have also reported higher engagement, presence, perception, emotions, empathy, learning stimulation, and enjoyment^11,12,19–22^. Moreover, a recent study by Buchman and Henderson, which showcased multidisciplinary participants, even showed positive learning effects on communication competencies^22^.

Few articles discuss teaching procedural skills in pediatrics using 360-degree VR videos^23^. Curran et al. in 2021 studied the use of 360-degree VR videos in NR. In this qualitative study based on descriptive phenomenology, they recruited 36 healthcare providers to view 360-degree VR videos relevant to their training. Using thematic analysis, they reported high level of acceptance and interest in the technology with benefits including a strong sense of presence and immersion.^23^. This exploratory study sets out to develop a 360-degree VR video capturing NR simulation, incorporating elements of AR inspired overlays to enhance the experience and game-like interactions. We also aim to determine its acceptability as an adjunct to teach NRP, all the while exploring aspects of NR that could benefit from it.

## Methods

### Setting and population

Between 09/2022 and 03/2023, three groups of healthcare providers were recruited at the CHU Sainte-Justine, a tertiary care mother–child hospital affiliated with the University of Montreal, which houses a 65-bed Neonatal Intensive Care Unit (NICU). Group 1 included 10 new NRP trainees with no previous NRP experience and attending the summer 2022 NRP training. Group 2 included 15–20 NRP providers with > 1 year and ≤ 5 years of experience. Group 3 consisted of 15–20 NRP providers with > 5 years of experience. The protocol was approved by the ethics review board of the CHU Sainte-Justine. Informed consent was obtained and documented from all participants. All research was performed in accordance with the guidelines set forth by the Hospital’s ethics board. Consent for publication was obtained from participants in the video.

### Study design

**Figure 1:**
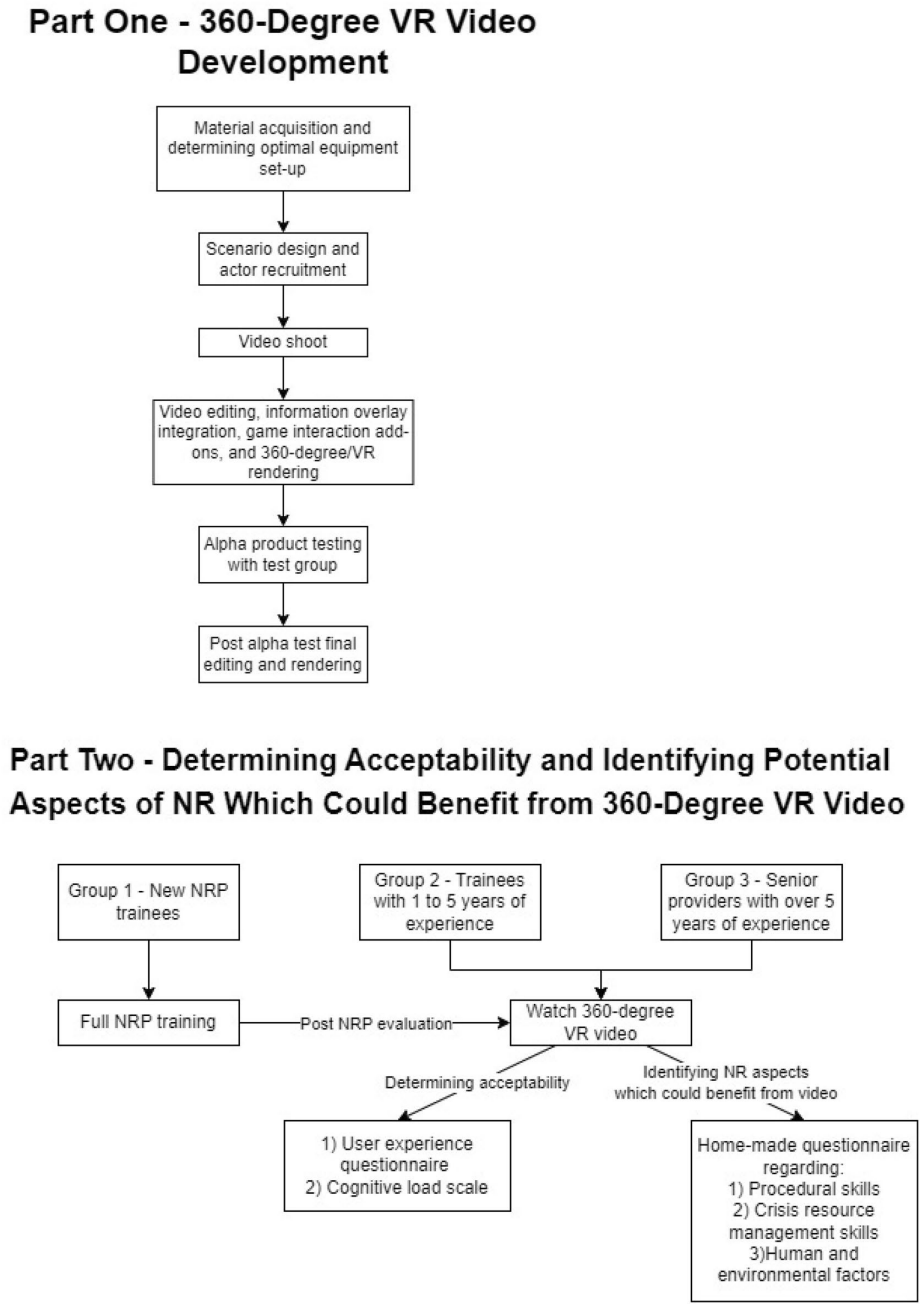
Study design flow charts for the two phases of the study.

This exploratory development study was divided in two phases (Fig. 1).

#### Phase 1—360-degre VR video development

The development of the 360-degree VR video was based on elements which the literature has shown as being positive for learning. First, effective characteristics of three-dimensional virtual learning environments and VR were taken into consideration: spatial knowledge representation, experiential learning, engagement, contextual learning, collaborative learning, situational learning, emotional experiences, and place illusion^24,25^. Second, shorter videos have been shown to maintain learners’ engagement more effectively^26^. Third, the more a HMD can produce immersion, the greater it will induce place illusion, positive affect, and will lead to better learning outcomes. More immersive devices also demonstrate lower prevalence of simulator sickness (nausea, headache, etc.)^24^. Fourth, the immersion produced with the HMD coupled with the immersiveness of the video itself (vivid graphics, stereoscopic sound), are not only more compelling for the learner, but also increases the amount of auditory information recall^19,24^. Finally, game-based learning environments are most effective to improve learning outcome gains and if the learner has the opportunity to practice a concept that they already learnt via another method, the gains are even greater^27^.

The following material was required: a powerful computer, including an 11th generation Intel® Core™ i9 Processor and a NVIDIA GeForce RTX™ 3070 GPU; a GoPro MAX™ 6K 360° action camera; Adobe Premiere Pro, Unity Editor; a complete resuscitation room will all the equipment necessary for resuscitation; a high-fidelity neonatal manikin; and a team of healthcare professionals (leader, airway management, heart rate and hemodynamics monitoring, nurse assigned to equipment, and nurse assigned to take notes). The projection of the video required a powerful computer, including an Intel Core i9 processor and a NVIDIA RTX 3070 graphics card, a HMD, in our case, an Oculus Rift S and a dedicated viewing room in the NICU.

**Figure 2:**
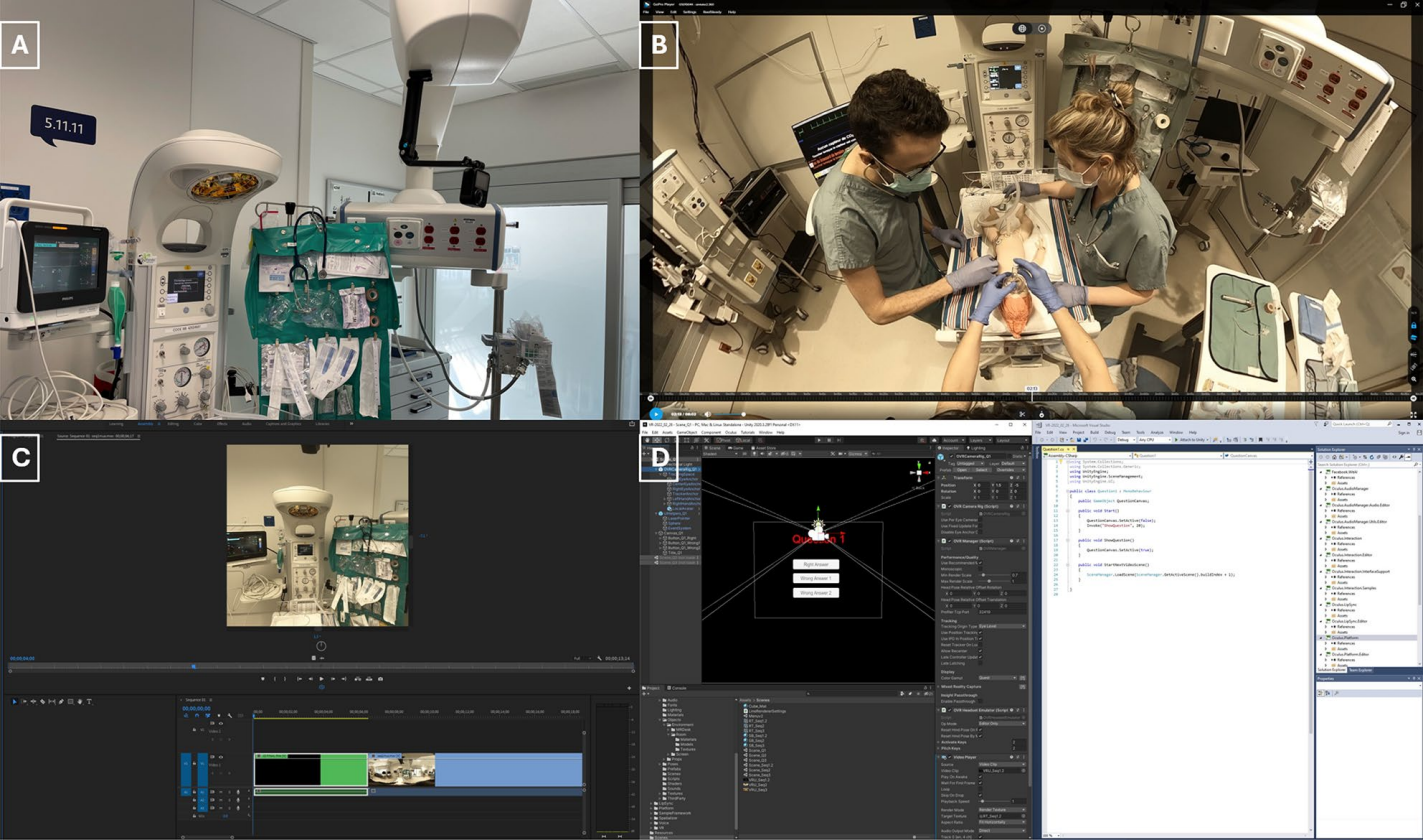
Development process. (**A**) Simulation room setup with placement of the 360-degree camera. (**B**) Initial footage of the 360-degree video viewed through the GoPro Player. (**C**) Editing process in Adobe Premier Pro. The program allows to edit a 360-degree video in a rectangular format. (**D**) Adding the VR interface and coding the game interactive questions using Unity Editor.

After having acquired all necessary technological material, tests were completed with the GoPro MAX™ 6K 360-degree action camera to determine its optimal placement. We chose to capture the resuscitation from the point of view of the resuscitator at the head of the neonate as this provided an overview of the team, the resuscitation, and the procedures at all times. We placed the camera on the room’s mechanical arm to achieve this (Fig. 2A). Parallel to these, a scenario was developed to take the viewer through the complete NRP 8^th^ edition algorithm and the necessary volunteer actors were recruited. While designing the video, incorporation of game-based interactive learning elements, which have shown to have positive effects on learning outcomes, was considered^27^. Furthermore, enhancement of the *immersivness* of the video was reached using a high-resolution graphics card and a HMD.

The video was divided into two sections and lasted 15 min. Through a narrated walkthrough and AR inspired overlaid informational cues, the first section introduced the resuscitation team and room, including the equipment present. The second section of the video consisted of the resuscitation itself segmented according to the resuscitation steps of the NRP algorithm.

**Figure 3:**
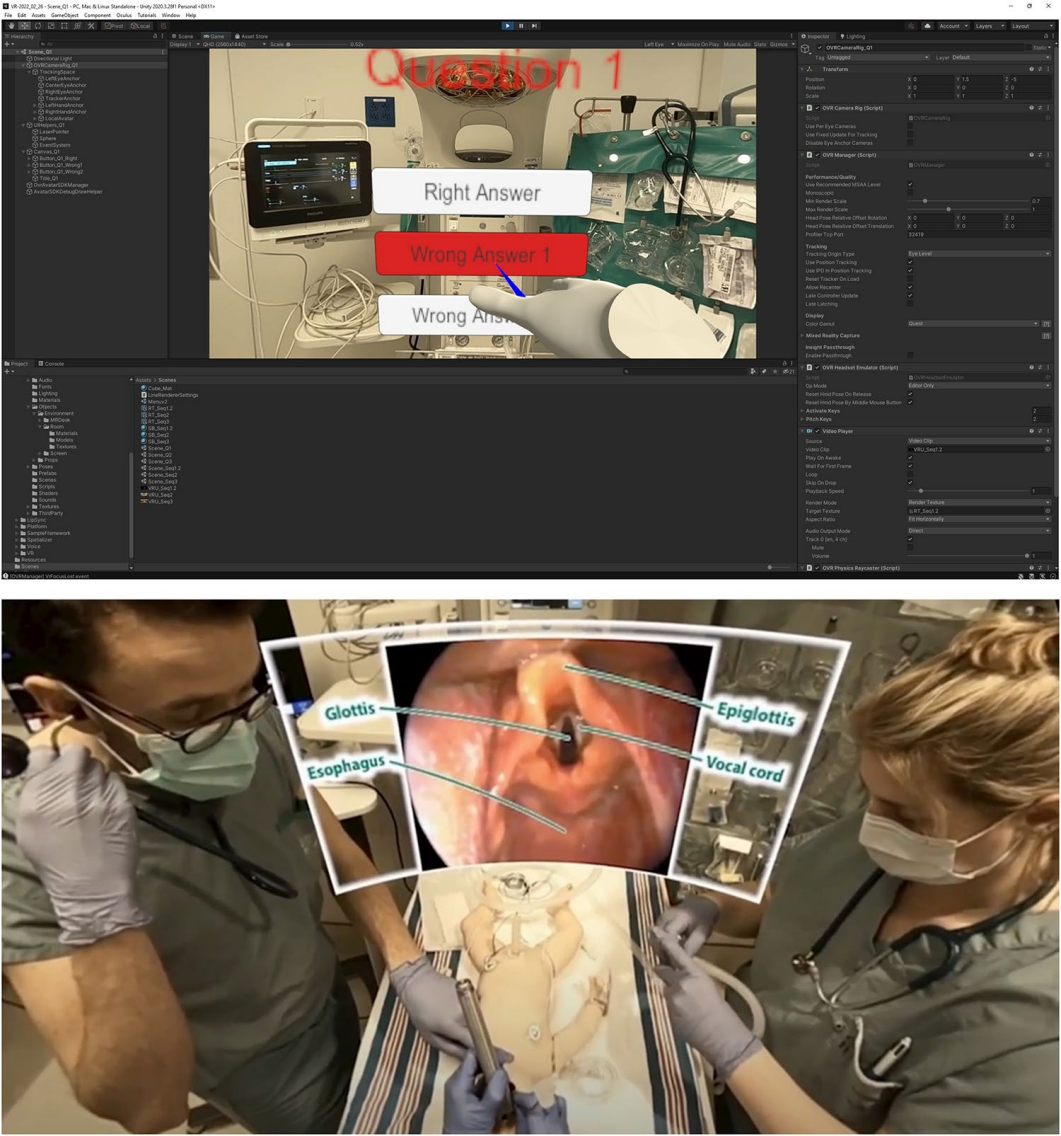
Interactive elements and overlaid informational cue. Top: testing the interactive questions using Unity Editor. Bottom: an example of an informational cue, here, during the endotracheal intubation process.

After the video shoot was completed, it was edited using Adobe Premiere Pro, as well as the GoPro Player. Game-like interactions and projection in VR were made possible through the Unity Editor. At key moments of the resuscitation (i.e., equipment preparation, ventilation management, etc.), interactions in the form of questions were incorporated, which participants needed to correctly answer, using the Oculus controllers, to progress through the video. An “alpha product test” was conducted after the editing with participants not included in the study. This allowed us to get initial feedback on the experience and detect any editing errors. Figure 2 depicts the main steps of the video’s development process, while Fig. 3 depicts some of the overlaid informational cues.

#### Phase 2—acceptability of 360-degree VR video

The second part of the study consisted in determining if the 360-degree VR video was an acceptable adjunct to teach NRP and to determine which aspects of NR could benefit from its incorporation in the NRP. Group 1 watched the video after the completion of their NRP training and groups 2 and 3 watched it at recruitment.

### Data collection

Participants answered four different questionnaires after experiencing the 360-degree VR video (Supplemental Materials). All questionnaires included Likert-type scale questions.

(Q1): Demographic questionnaire collected data on healthcare provider role, years of NRP experience, and previous experience with VR.
(Q2): User experience questionnaire, adapted by Tcha-Tokey et al.^28^. This is a validated and unified questionnaire on user experience in immersive virtual environments. It contains 9 subscales that best define user experience: presence, engagement, immersion, flow, skill, emotion, experience consequence, judgment, and technology adoption. We modified wording in some questions to be more appropriate for 360-degree VR videos (e.g. we took out hardware terms that were not used such as “keyboard”, “gamepad”, “cross hair”, and we took out action words that were not relevant such as “moving around”). We also modified wording in the “emotions” subscale to optimize statistical analysis by uniformizing emotions (e.g. “I did not feel nervous” and “I did not feel like distracting myself” to match the positive wording used in the rest of the questions such as “enjoyed”, “excited”. All modifications are presented in Supplemental Materials.). The questionnaire has a Likert scale from 1 to 10 (1: strongly disagree and 10: strongly agree) for eight of the subscales, a differential scale for one of the subscales, and three open-ended questions.
(Q3): Adapted version of the Paas cognitive load (CL) scale^29^. This short subjective cognitive load scale evaluates cognitive load as a whole on a Likert scale from 1 to 9 (1: very very low mental effort and 9: very very high mental effort). Group 1 answered this questionnaire three times, once for each segment of the NRP training (self-directed and in-person) and once for the 360-degree VR video. Groups 2 and 3 answered this questionnaire only once for the 360-degree VR video.
(Q4): Home-made questionnaire to identify aspects of NR which could benefit from the incorporation of 360-degree VR videos in the NRP training program. By asking “After having experienced the immersive 360-degree video, I believe it can help in teaching … (e.g. Positioning the airways, Administering ET or IV epinephrine, etc.)” we specifically emphasized procedural skills, crisis resource management skills, and human factors. It also contained one general appreciation question. The questionnaire has a Likert scale from 1 to 5 (1: strongly disagree and 5: strongly agree). This questionnaire was conceived by targeting general resuscitation skills, as well as the procedural skills of NR. It was piloted by two healthcare professionals experienced in NR who agreed on their pertinence.

### Statistical analysis

Data was collected using REDCap survey tool and analysis was completed using SPSS (V25). Descriptive data was presented using means and standard deviations. Comparisons between groups from the user experience, cognitive load, and homemade Likert scale questionnaires were done using one-way ANOVA. The user experience questionnaire uses a 10-point Likert scale to quantify the participants’ perception of the subscales mentioned above. For the seven positive subscales (presence, engagement, immersion, flow, emotion, skill, and technology adoption), the higher the score of a component, the better it is perceived by the user. Inversely, for the experience consequence subscale, a lower score quantifies a better user experience. 5 being the neutral score, acceptability of the 360-degree VR video as a simulation method was thus defined as a median ≥ 6 for the positive subscales and ≤ 4 for the negative subscale. The final subscale, judgement, being on a differential scale of 1 vs 2, a mean score of > 1 was deemed acceptable. The aspects of NR that participants identified as having a potential to benefit from the 360-degree VR video scored a mean ≥ 3. For group 1, we compared cognitive load results between NRP training segments and the 360-dgree VR video using a one-way ANOVA analysis. We also calculated Cronbach-Alpha values of our user experience questionnaire utilisation and compared performance with Tcha-Tokey et al. original study’s values. Finally, we performed a qualitative thematic analysis of the open-ended questions of the user experience questionnaire.

## Results

### Phase 1

Using the aforementioned technological material, our team was able to create a high quality 360-degree VR video combining the benefits of 360-degree videos, which offer no interactive elements, but true representation of reality, with VR, which offers interaction capabilities, but a virtual representation of reality. We were successful in integrating game interactions in the form of interactive questions and overlaid informational cues (e.g. intubation table, chest compression schematics, MR. SOPA prompts). In addition, this novel video is in French and showcases neonatal resuscitation simulation including elements of Crisis Resource Management, and Human and Environment Interactions. Furthermore, the Unity framework we built requires minimal modifications to entirely swap the video segments and interactive elements, allowing easy conception of new simulation environments. The following link allows to view a short, accelerated clip: https://drive.google.com/file/d/1M95s-jRGnxB9dX9qqJsQNuVDYKq5P3W8/view?usp=sharing. The full 15-min video can be accessed upon request to the authors on the Unity Editor platform.

### Phase 2

Of the 48 healthcare professionals approached to participate in the study, 46 were recruited. Ten were new trainees, 16 had between 1 and 5 years of NRP experience, and 20 had over 5 years of NRP experience (Table 1). There was a significant difference between the profession of participants and their NRP experience, such as the less experienced ones were mostly pediatrics residents (p = 0.02). There was no difference between those with and without any VR experience.

**Table 1:** Participants according to healthcare professional status, virtual reality experience and NRP experience.

Participating healthcare professionals	Participant category—NRP experience (years)			p
	Group 1 < 1	Group 2 Between 1 and 5	Group 3 > 5	
Pediatrics Resident	8	11	1	0.02
Neonatology Nurse Practitioner	2	2	4	
Neonatologist	0	0	7	
Neonatology Clinician Nurse	0	1	4	
Neonatology Fellow	0	2	2	
Respiratory Therapist	0	0	2	
Any Virtual Reality Experience	2	5	6	0.8

Results comparing the performance of the user experience questionnaire in our study to the original study are presented in Table 2. All Cronbach’s alphas, except one, were acceptable, and most were stronger than the original study.

**Table 2:** User experience questionnaire results by subscales: mean values presented with their standard deviations, with Cronbach’s alpha comparison between our study and the original study.

User experience subscale	NRP experience category			p value	Original Cronbach’s Alpha	Study Cronbach’s Alpha
	Group 1 < 1 year	Group 2 > 1 year, < 5 years	Group 3 > 5 years			
Presence	8.01 (1.13)	7.11 (1.52)	7.33 (1.97)	0.40	0.755	0.931
Engagement	8.1 (1.52)	7.54 (1.49)	7.3 (2.26)	0.55	0.759	0.845
Immersion	7.36 (2.37)	6.83 (1.78)	5.87 (2.31)	0.17	0.767	0.887
Flow	7.01 (1.13)	6.38 (1.51)	6.33 (1.81)	0.52	0.826	0.838
Emotion	7.38 (1.12)	6.67 (1.23)	6.26 (2.10)	0.23	0.718	0.898
Skill	8.23 (1.33)	6.91 (1.70)	7.73 (1.61)	0.11	0.820	0.853
Experience Consequence	2.45 (1.33)	3.14 (1.81)	3.42 (2.16)	0.42	0.908	0.883
Technology Adoption	8.75 (1.34)	7.44 (1.73)	8.01 (1.87)	0.18	0.781	0.916
Judgment	1.97 (0.04)	1.96 (0.09)	1.96 (0.11)	0.82	0.804	0.588

Results of the user experience questionnaire are presented in Table 2. To the exception of the “immersion” mean of the most experienced participants, all means in the positive subscales were superior to 6 (presence, engagement, immersion, flow, emotion, skill, and technology adoption). All mean results in the singular negative subscale “experience consequence” were below 4. All mean results in the differential “judgment” subscale were above 1. There were no differences between participant categories.

The Paas CL means decreased with more NRP experience: 5.7 ± 1.49 for group 1, 5.81 ± 1.64 for group 2, and 4.95 ± 1.27 for group 3, but this was not significantly different (p = 0.17). When comparing the CL between NRP training segments for new trainees, the 360-degree VR video showed a significantly lower CL (5.7 ± 1.49) compared to the first two parts (textbook: 6.2 ± 1.23 and in-person session: 7.9 ± 0.87) of the NRP training (p < 0.01).

The general appreciation mean score for the 360-degree VR video was high without any significant difference between participant categories: 4.90 ± 0.31 for group 1, 4.69 ± 0.48 for group 2, and 4.4 ± 0.82 for group 3; p = 0.11. Results of the homemade questionnaire to identify aspects of NR which could benefit from the 360-degree VR video are presented in Table 3.

**Table 3:** Identification of aspects of NR which could benefit from the incorporation of 360° VR video in the NRP training program; mean values presented with their standard deviations.

Questionnaire category	Elements	NRP experience category			p value
		Group 1 < 1 year	Group 2 > 1 year, < 5 years	Group 3 > 5 years	
Procedural Skills	Primary evaluation of the neonate	5 (0)	4 (0.89)	4 (0.92)	< 0.01
	Positioning of the airway	4.3 (0.82)	4 (0.81)	3.9 (1.12)	0.56
	Suctioning and clearing the airway	4.5 (0.85)	4 (0.63)	3.8 (1.05)	0.13
	Positioning a mask to administer CPAP or PPV	4.5 (0.85)	3.81 (1.04)	3.75 (1.12)	0.16
	Ensuring adequate ventilation and performing corrective steps for PPV	4.8 (0.63)	4.13 (1.02)	3.75 (1.12)	0.03
	Performing endotracheal intubation	3.7 (0.67)	3.44 (0.96)	3.25 (1.02)	0.46
	Performing chest compressions, coordinated with ventilation	4.5 (0.85)	3.41 (0.99)	3.9 (0.91)	0.22
	Administering ET or IV epinephrine	4.4 (0.69)	3.88 (1.14)	3.9 (1.07)	0.39
Human and Environment Factors	Positioning according to role and other members	4.3 (0.95)	4.13 (0.95)	4.1 (0.64)	0.81
	Positioning according to equipment	4.2 (0.92)	4.19 (0.98)	4.15 (0.67)	0.98
	Identifying equipment and material in a resuscitation room	4.7 (0.48)	4.38 (0.72)	4.2 (0.61)	0.13
	Choosing equipment for initial steps	4.4 (0.51)	4.13 (0.80)	4 (0.65)	0.33
	Choosing equipment for ventilation	4.3 (0.95)	4 (0.73)	4.2 (0.52)	0.53
	Choosing equipment for intubation	4.3 (0.95)	4.13 (0.88)	4.15 (0.49)	0.83
Crisis Resource Management	Anticipating and planning	4.4 (0.69)	4.25 (0.57)	3.75 (1.02)	0.08
	Knowing the environment	4.6 (0.51)	4.44 (0.72)	4.25 (0.64)	0.36
	Designating leadership	3.5 (0.85)	3.81 (0.91)	3.05 (1.35)	0.13
	Distributing the workload	3.9 (0.87)	4 (0.89)	3.6 (0.99)	0.42
	Establishing role clarity	4.3 (0.67)	4.13 (0.88)	3.85 (0.87)	0.35
	Using all available information	4.8 (0.42)	4 (0.73)	3.75 (0.85)	< 0.01
	Allocating attention wisely	4.2 (0.63)	3.94 (0.68)	3.5 (1.05)	0.09
	Calling for help early	4.1 (0.99)	3.75 (1)	3.3 (0.98)	0.1
	Mobilizing resources	4.4 (0.96)	3.63 (0.88)	3.4 (1.18)	0.05
	Using cognitive aids	4.6 (0.51)	4 (0.63)	3.95 (0.68)	0.03
	Communicating effectively	4 (0.81)	3.94 (0.85)	3.45 (1.91)	0.24

Elements of the questionnaire categories were asked in the following format: “After having experienced the immersive 360-degree video, I believe that it can help in teaching …”.

The highest mean values are found in the “Human and Environment Factors” category, all of them being superior to 4 and not one element showed any statistically significant difference between participant categories. All means for the “Procedural Skills” and “Crisis Resource Management Skills” were superior to 3 and, respectively, only two and three elements showed a statistically significant difference between participant categories.

The main recurring themes of the open-ended questions of the user experience questionnaire are presented in Table 4.

**Table 4:** Summary of the main recurring comments following quantitative analysis.

	Positive remarks	Negative remarks	Suggestions
Themes	Realistic experience	Side effects of VR immersion	Adding interactive VR elements
	Immersion and interaction	No interaction with projected material and environment	Possibility to have changing POVs
	Academic potential	Quality of image	Possibility of adding voice interactive elements
	Stress-free practice	Video progresses too fast	Adding choice consequence

VR, virtual reality; POV, point of view.

## Discussion

Our first finding was that the development of a high quality 360-degree VR video in French, combining the benefits of classic 360-degree videos with VR and showcasing the NRP algorithm including elements of Crisis Resource Management, and Human and Environment Interactions was possible. Incorporating game-like interactions is also feasible, using hardware and software readily available to the public. Only one other study has published a developed 360-degree VR video in NR^23^. Based on our experience, we highly recommend using Adobe Premiere Pro for editing. Unity Editor proved to be a challenging program to use, but ultimately it allowed us to develop the video we had envisioned. The created framework also allows us to seamlessly produce new virtual simulation environments. Research teams and educators should identify what they want to achieve with the video prior to embarking on learning Unity’s interface and functionalities. We were however faced with technical limitations, such as using a budget friendly 360-degree camera that did not fully deliver in terms of camera functionality, picture quality, and sound recording. As a matter of fact, when switching to the 360-degree mode, the GoPro MAX™ 6K 360° action camera lost its 6K resolution. Also, its stereoscopic sound could not be extracted using post-marketing tools.

Patel et al., in their tutorial article for developing 360-degree VR videos for trauma, and O’Sullivan et al., in their tutorial article for developing low-cost 360-degree VR videos present a set of recommendations to efficiently create such videos. After careful consideration, Patel et al.’s team ended up using YI Technology 360-degree VR cameras, while O’Sullivan’s team explored Ricoh Theta S and Samsung Gear 360 models; the latter team found earlier versions of GoPro cameras disadvantageous. Both teams used external microphones to reliably record voices and have higher immersion with stereoscopic sounds and, later, an Adobe Premier software as their editing software^10,30^. Based on the difficulties and technological shortcomings we faced, we concur with them to select proper microphones for stereoscopic sound addition. Built-in microphones in 360-degree cameras lack in quality, especially when recording in an environment with lots of ambient noise. We also agree to the importance of picking a camera that suits the video quality needed. Finally, Patel et al.^10^ suggest finding production partners to further elevate the quality of future videos, by having additional experts’ inputs on development and editing.

The aforementioned tutorials do not discuss the addition of overlays elements. Adobe Premiere Pro and Adobe After Effects seem to be the most used software for such additions. Recent studies exploring neurosurgical and operative 360-degree VR videos, central line insertion 360-degree VR videos, and development of such videos to portray experience of migraine sufferers were all successful in adding these elements through one or both of the Adobe software^13,21,31,32^. Harrington et al.^21^ were also successful in using Mettle Skybox Studio Plugin in their 360-degree operative VR videos. In our literature review, Chao et al.^14^ were one of the only ones to use Unity Editor to develop and add their courseware to their 360-degree video to history taking and physical examination.

Our second finding is that the 360-degree VR video is an acceptable simulation method for healthcare professionals of different clinical experience. Indeed, the user experience results show overall high means in the positive subscales and a low mean in the “experience consequence” subscale. The “judgment” subscale’s results with differential questions shows high appreciation. The cognitive load is at an acceptable level showing a lower level than other forms of education for novice trainees, and the general appreciation of all participants is high. Only one other study has looked at the use of 360-degree VR video in NR and reported, although only with qualitative results, high level of acceptance of VR headsets and 360-degree VR videos, a strong sense of presence and immersion, greater level of interest and its value as a supplemental teaching resource^23^.

The theory of CL represents the limit of a learner’s capacity to process information and is an important consideration while designing multimedia teaching material^33,34^. CL includes three distinct elements: intrinsic (inherent complexity of the task), extraneous (superfluous processes that do not directly contribute to learning), and germane load (mental process representing the actual learning)^34,35^. Our results show CL scores < 6 and the most experienced group had the only mean score < 5. During the design of the scenario, the shooting, as well as the editing of the video, we applied concepts described by Mayer and Moreno to reduce the CL of this video. To name a few, these included segmenting (allowing a short break between video segments), synchronizing (presenting narration and corresponding animation simultaneously), and weeding (eliminating extraneous material)^34^. Studies assessing the CL of VR technologies show conflicting results. Frederiksen et al. showed that immersive VR laparoscopy training induced a higher CL and resulted in a poorer performance compared to non-immersive VR training. They hypothesized that a higher degree of immersion and the novelty of the technology resulted in a higher intrinsic and extraneous load; repeated exposure, however, diminishes the CL^35^. Pirker and Dengel also noted CL as being one of the disadvantages of these immersive technologies^11^. However, Yi-Ping Chao et al. did not discern a higher CL using the Paas CL scale and the NASA Task Load Index while comparing 360-degree VR videos to 2D videos which taught history taking and physical examination skills^14^. We ask ourselves if robust prior experience in NR possibly contributed to lowering the intrinsic and germane load for our senior participants. Interestingly, new trainees reported a significantly lower CL when compared to in-person training and textbook learning. It is possible that these participants viewed the 360-degree VR video as a stress and evaluation free learning environment, and less of a workload than a textbook. We can also hypothesize that they were less comfortable with in-person simulations at the beginning of their training leading to a higher cognitive load.

Finally, our last finding is that these types of videos could be effective for teaching technical skills, crisis resource management principles, and human–environment interactions. The mean scores were highest for the human–environment interactions, and it was also the only category in which none of the components had any significant difference between participants. When looking at the overall results, we notice that scores are generally higher the more novice the participant is.

Our study presents some limitations. We expect that a selection bias, more specifically a volunteer bias, might have favorably influenced our results due to the fact that the sampling was done entirely in our department. Furthermore, an ascertainment bias due to the novelty of the technology may have led to a response bias. Other possible response biases in our study are inherent to the Likert scale type questionnaires. The main limitation of this study’s results is in the nature of the study design itself. Being an exploratory study, there is an absence of clearly measured learning outcomes, both short and long term. Furthermore, there are few literature-validated resources available for 360-degree VR videos. This led us to develop and use a home-made questionnaire to evaluate aspects of NR which could benefit from this simulation tool. We were thus incapable to compare categories between themselves to determine if one is statistically more significant.

## Conclusions

360-degree VR videos could potentially be new simulation adjuncts and teaching material for the NRP, especially as supplements to the self-directed portion. Further studies to evaluate short and long-term learning outcomes, and to assess the CL of VR technologies are required. Future considerations include creating additional scenarios, developing videos with switchable point of views, adding additional interactive elements, and adding choice consequence elements (i.e., not having a linear progression, but rather having the participant’s answers determine the outcome of the resuscitation).

### Supplementary Information

Supplementary Information.

## Data Availability

Deidentified individual participant data (including data dictionaries) will be made available, in addition to study protocols, the statistical analysis plan, and the informed consent form. The data will be made available upon publication to researchers who provide a methodologically sound proposal for use in achieving the goals of the approved proposal. Proposals should be submitted to ahmed.moussa@umontreal.ca.
