# Supplementary material for: 360-degree virtual reality video to teach neonatal resuscitation: an exploratory development study
Source: Sci Rep. 2024 Jun 22;14:14383. doi: 10.1038/s41598-024-65299-4 (PMC11193781; doi:10.1038/s41598-024-65299-4)
Supplement: Supplementary file 1 — Supplementary Information. [file 41598_2024_65299_MOESM1_ESM.docx]

**360-Degree Virtual Reality Video to Teach Neonatal Resuscitation: An Exploratory Development Study.**

**Supplemental Materials**

Tachejian Sevag, MD^1,2^, Moussa Ahmed*, MD, MMEd^3,4,5^

**Affiliations:**

^1^Department of Pediatrics, Division of Pediatric Emergency Medicine, The Hospital for Sick Children, Toronto, Ontario, Canada.

^2^Department of Pediatrics, Division of General Pediatrics, CHU Sainte-Justine, Montreal, Quebec, Canada.

^3^Department of Pediatrics, Division of Neonatology, CHU Sainte-Justine, Montreal, Quebec, Canada.

^4^CHU Sainte-Justine Research Center, Montreal, Quebec, Canada.

^5^Centre for applied health sciences education (CPASS), Faculty of Medicine, Université de Montréal, Montreal, Quebec, Canada.

**Corresponding author:**

Ahmed Moussa

Department of Pediatrics, University of Montreal

CHU Sainte-Justine

3175 Chemin Cote Sainte-Catherine

Montreal (QC), H3T 1C5.

Tel: 514-345-4931

Email: [ahmed.moussa@umontreal.ca](mailto:ahmed.moussa@umontreal.ca)

https://orcid.org/0000-0002-2556-6970

**User experience questionnaire**

Adapted from Tcha-Tokey et al.

*All items are on a 10-point Likert scale, except open ended questions and differential scale items.*

*Strikethrough and bold words represent our adaptations.*

| **Item #** | **Item** | **Subscale** |
| --- | --- | --- |
| 1 | My interactions with the virtual environment seemed natural | Presence |
| 2 | The visual aspects of the virtual environment incited me to get involved | Engagement |
| 3 | The devices ~~(gamepad or keyboard)~~ which controlled my movement in the virtual environment seemed natural | Presence |
| 4 | I was able to actively survey the virtual environment using vision | Presence |
| 5 | The sense of ~~moving~~ **looking around** ~~inside~~ the virtual environment was compelling | Engagement |
| 6 | I was able to examine objects closely | Presence |
| 7 | I could examine objects from multiple viewpoints | Presence |
| 8 | I was involved in the virtual environment experience | Engagement |
| 9 | I felt proficient in ~~moving and~~ interacting with the virtual environment at the end of the experience | Presence |
| 10 | I could concentrate on the assigned tasks rather than on the devices (gamepad or keyboard) | Presence |
| 11 | I correctly identified sounds produced by the virtual environment | Presence |
| 12 | I correctly localized sounds produced by the virtual environment | Presence |
| 13 | I felt stimulated by the virtual environment | Immersion |
| 14 | I became so involved in the virtual environment that I was not aware of things happening around me | Immersion |
| 15 | I became so involved in the virtual environment that it felt as if I was inside the game rather than manipulating a gamepad and watching a screen | Immersion |
| 16 | I felt physically fit/well in the virtual environment | Immersion |
| 17 | I became so involved in the virtual environment that I lost all track of time | Immersion |
| 18 | I felt I could perfectly control my actions | Flow |
| 19 | At each step, I knew what to do | Flow |
| 20 | I felt I controlled the situation | Flow |
| 21 | Time seemed to flow differently than usual | Flow |
| 22 | Time seemed to speed up | Flow |
| 23 | I was losing the sense of time | Flow |
| 24 | I was not worried about what other people would think of me | Flow |
| 25 | I felt I was experiencing an exciting moment | Flow |
| 26 | This experience was giving me a great sense of well-being | Flow |
| 27 | When I mention the experience in the virtual environment, I feel emotions I would like to share | Flow |
| 28 | I enjoyed being in this virtual environment | Emotion |
| 29 | It was so exciting that I could stay in the virtual environment for hours | Emotion |
| 30 | I enjoyed the experience so much that I feel energized | Emotion |
| 31 | I **did not feel** ~~felt~~ nervous in the virtual environment | Emotion |
| 32 | I **did not feel** ~~felt~~ like distracting myself in order to reduce my anxiety | Emotion |
| 33 | I found my mind wandering while I was in the virtual environment | Emotion |
| 34 | The interaction devices (Oculus headset, gamepad and/or keyboard) **did not** bore~~d~~ me to death | Emotion |
| 35 | When my actions were going well, it gave me a rush | Emotion |
| 36 | While using the interaction devices (Oculus headset, gamepad and/or keyboard), **I did not feel** ~~felt~~ like time was dragging | Emotion |
| 37 | I enjoyed the challenge of learning the virtual reality interaction devices (Oculus headset, gamepad and/or keyboard) | Emotion |
| 38 | I enjoyed dealing with the interaction devices (Oculus headset, gamepad and/or keyboard) | Emotion |
| 39 | I felt confident selecting objects in the virtual environment | Skill |
| 40 | I felt confident moving the ~~cross-hair~~ **hands** around the virtual environment | Skill |
| 41 | I felt confident using the gamepad ~~and/or keyboard~~ to ~~move~~ **look** around the virtual environment | ~~Skill~~ |
| 42 | I feel confident understanding the terms/words relating to the interaction devices (Oculus headset, gamepad and/or keyboard) | Skill |
| 43 | I feel confident learning advanced skills within a specific virtual reality software using the Oculus headset | Skill |
| 44 | I feel confident describing the functions the interaction devices (Oculus headset, gamepad and/or keyboard, joystick, etc.) of a virtual reality environment | Skill |
| 45 | Personally, I would say the virtual environment is impractical/practical | Judgement |
| 46 | Personally, I would say the virtual environment is confusing/clear | Judgement |
| 47 | Personally, I would say the virtual environment is unruly/manageable | Judgement |
| 48 | I found that this virtual environment was lame/exciting | Judgement |
| 49 | I found this virtual environment amateurish/professional | Judgement |
| 50 | I found this virtual environment gaudy/classy | Judgement |
| 51 | I found this virtual environment unpresentable/presentable | Judgement |
| 52 | I found that this virtual environment is ugly/beautiful | Judgement |
| 53 | I found that this virtual environment is unlikeable/likeable | Judgement |
| 54 | I suffered from fatigue during my interaction with the virtual environment | Experience consequence |
| 55 | I suffered from headache during my interaction with the virtual environment | Experience consequence |
| 56 | I suffered from eyestrain during my interaction with the virtual environment | Experience consequence |
| 57 | I felt an increase of my salivation during my interaction with the virtual environment | Experience consequence |
| 58 | I suffered from nausea during my interaction with the virtual environment | Experience consequence |
| 59 | I suffered from fullness of the head during my interaction with the virtual environment | Experience consequence |
| 60 | I suffered from dizziness ~~with eye open~~ during my interaction with the virtual environment | Experience consequence |
| 61 | I suffered from vertigo during my interaction with the virtual environment | Experience consequence |
| 62 | If I use the same virtual environment again, my interaction with the environment would be clear and understandable for me | Technology adoption |
| 63 | It would be easy for me to become skillful at using the virtual environment | Technology adoption |
| 64 | Learning to operate the virtual environment would be easy for me | Technology adoption |
| 65 | Using the interaction devices (Oculus headset, gamepad and/or keyboard) is a ~~bad~~ **good** idea | Technology adoption |
| 66 | The interaction devices (Oculus headset, gamepad and/or keyboard) would make work more interesting | Technology adoption |
| 67 | I would like to work with the interaction devices (Oculus headset, gamepad and/or keyboard) | Technology adoption |
| 68 | I have the resources necessary to use the interaction devices (Oculus headset, gamepad and/or keyboard) | Technology adoption |
| 69 | In your opinion, what were the positive points about your experience? | N/A |
| 70 | In your opinion, what were the negative points about your experience? | N/A |
| 71 | Do you have suggestions to improve this virtual reality environment? | N/A |
